# Supplementary figures and images for: Wbm0076, a candidate effector protein of the Wolbachia endosymbiont of Brugia malayi, disrupts eukaryotic actin dynamics
Source: PLoS Pathog. 2023 Feb 17;19(2):e1010777. doi: 10.1371/journal.ppat.1010777 (PMC9980815; doi:10.1371/journal.ppat.1010777)

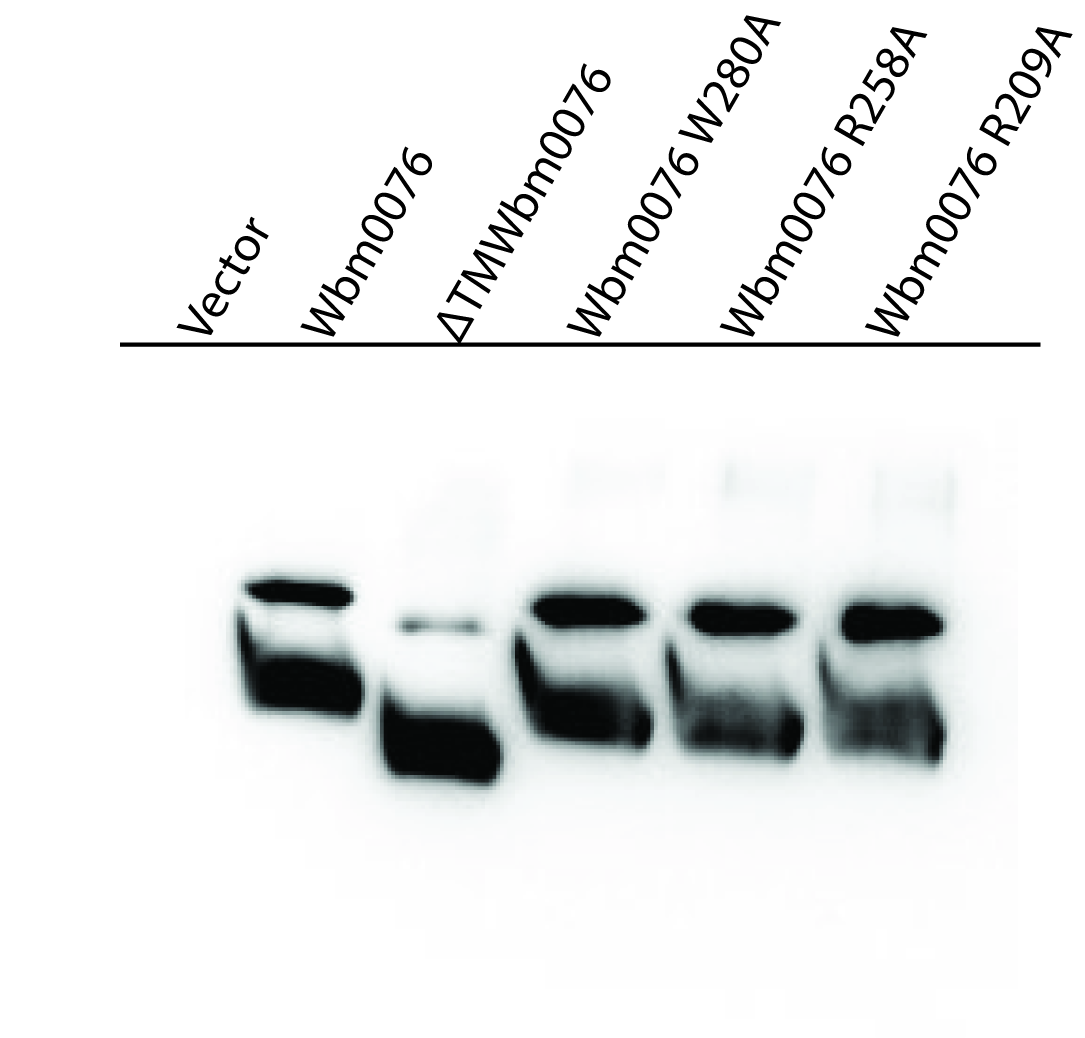

Supplement: S1 Fig — Yeast strains harboring a pYES2/NT A control plasmid, or a pYES2/NT A plasmid cloned with one of the following: wBm0076, ΔTMwBm0076, wBm0076 (W280A), wBm0076 (R258A), wBm0076 (R209A) were grown overnight in CSM medium lacking uracil. Cells were subcultured to fresh CSM-Ura containing or lacking 1 μM β-estradiol. After 5 h outgrowth at 30°C, cells were lysed, boiled in SDS-PAGE loading buffer, and loaded for western blot analysis. Anti-Xpress antibodies were used to probe the presence of each protein. (TIF) [file ppat.1010777.s001.tif]

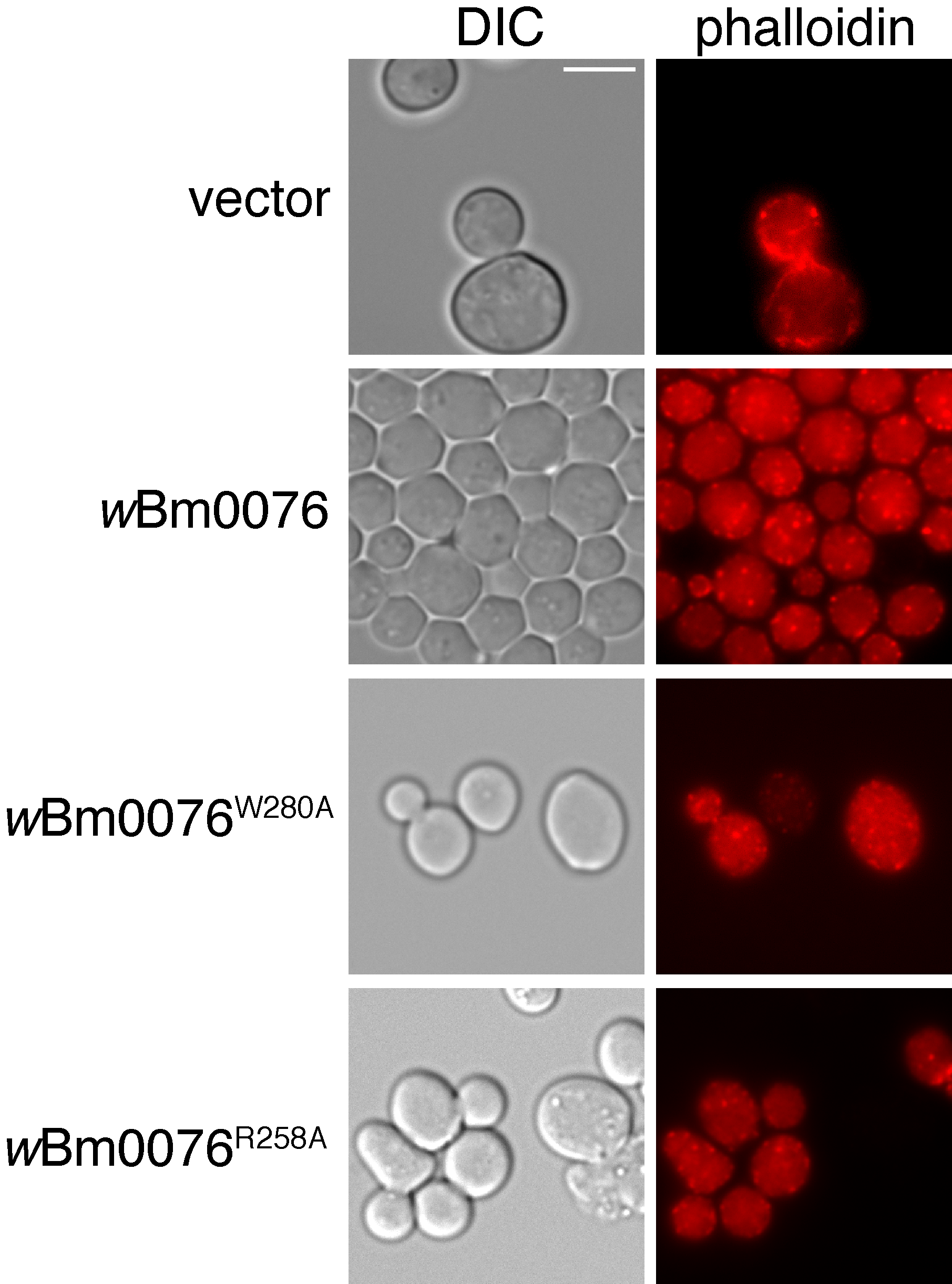

Supplement: S2 Fig — Yeast strains harboring a pYES2/NT A control plasmid, or a pYES2/NT A plasmid cloned with one of the following: wBm0076, wBm0076 (W280A), wBm0076 (R258A), were grown overnight in CSM medium lacking uracil. Cells were subcultured to fresh CSM-Ura and outgrown for 2h at 30°C with shaking. The actin cytoskeleton was stained with rhodamine phalloidin (S1 Methods) and imaged; bar = 5 μ. (TIF) [file ppat.1010777.s002.tif]

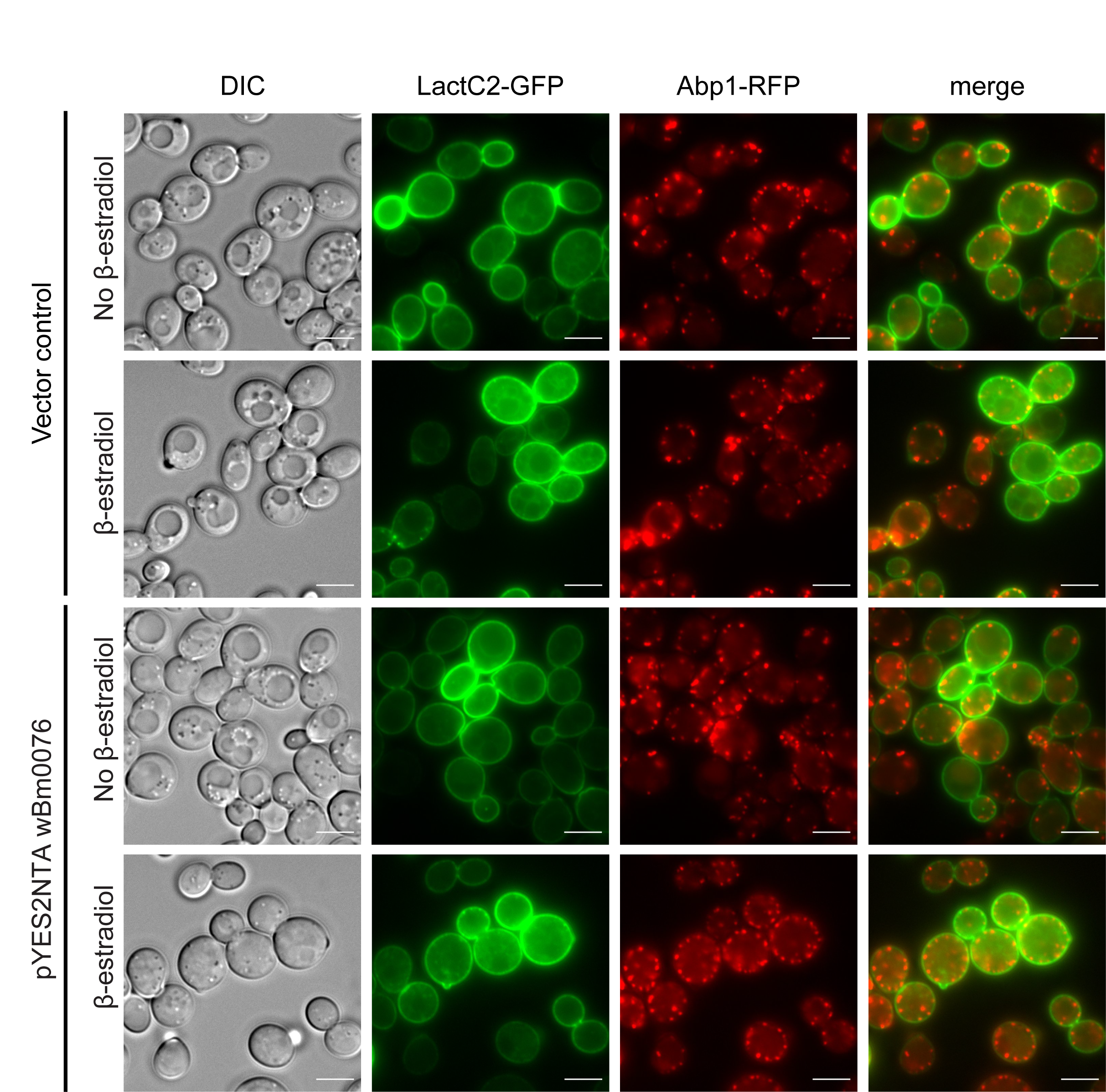

Supplement: S3 Fig — Yeast strains modified with GEV for β-estradiol-dependent induction of GAL promoters (Methods), expressing both Abp1RFP and LactC2-GFP, and harboring either a pYES2/NT A control plasmid or a pYES2/NT A plasmid cloned with wBm0076 were grown overnight in CSM medium lacking uracil. Cells were subcultured to fresh CSM-Ura containing or lacking 1 μM β-estradiol. After 5 h outgrowth at 30°C, cells were harvested and visualized. Bar = 5 μ. (TIF) [file ppat.1010777.s003.tif]

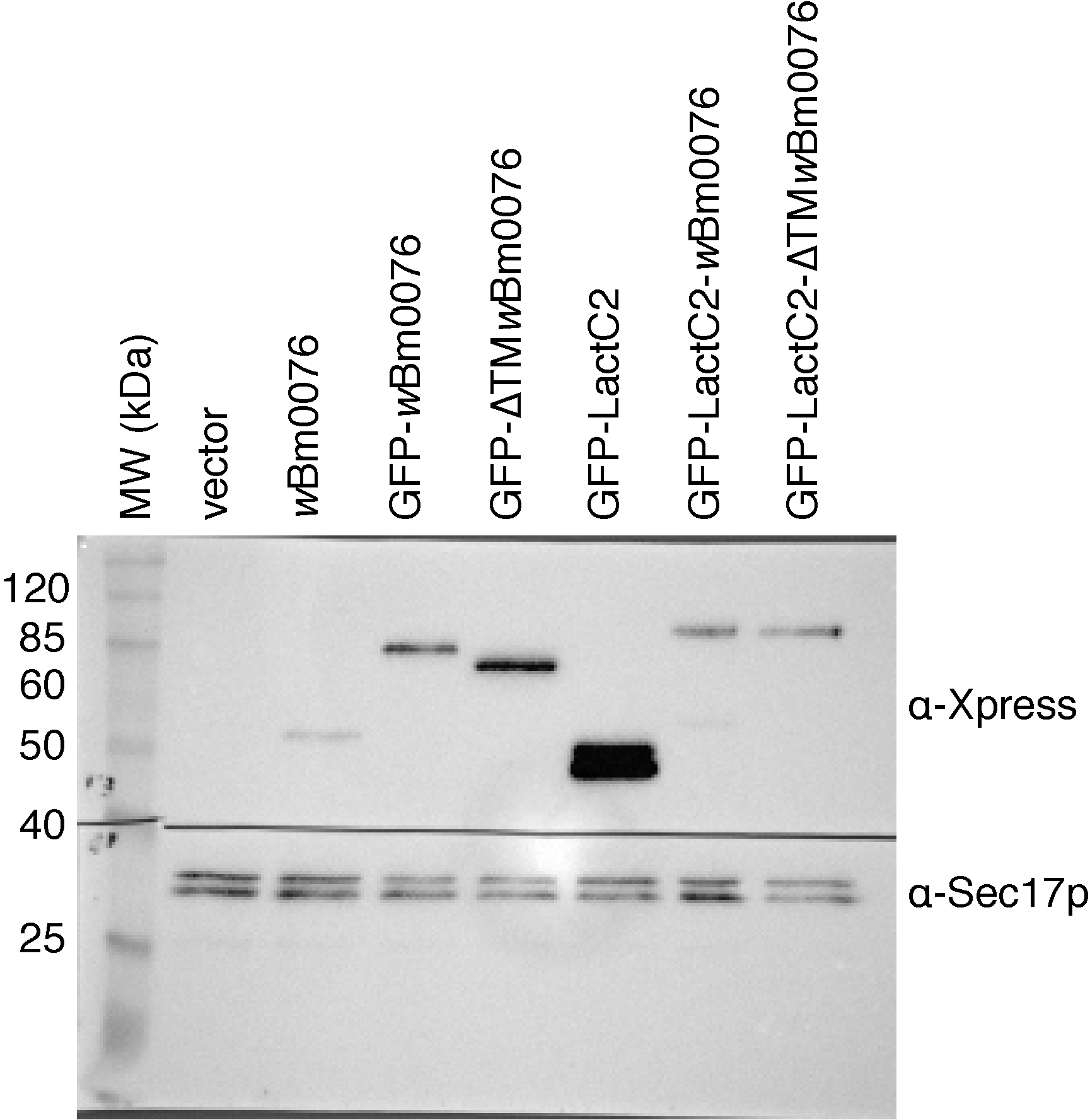

Supplement: S4 Fig — Yeast strains expressing Abp1-RFP and harboring either a pYES2/NT A control plasmid, or a pYES2/NT A plasmid cloned with one of the following: wBm0076, GFP-wBm0076, GFP-ΔTMwBm0076, GFP-LactC2, GFP-LactC2-wBm0076, or GFPLactC2-ΔTMwBm0076, were grown overnight in CSM medium lacking uracil. Cells were subcultured to fresh CSM-Ura containing or lacking 1 μM β-estradiol. After 5 h outgrowth at 30°C, cells were lysed, boiled in SDS-PAGE loading buffer, and separated via SDS-PAGE. After protein transfer, the membrane was bisected at ~40 kDa and the corresponding antibodies were used to detect wBm0076 derivatives (anti-Xpress) or the Sec17p loading control (anti-Sec17p). (TIF) [file ppat.1010777.s004.tif]
