# Supplementary material for: Wbm0076, a candidate effector protein of the Wolbachia endosymbiont of Brugia malayi, disrupts eukaryotic actin dynamics
Source: PLoS Pathog. 2023 Feb 17;19(2):e1010777. doi: 10.1371/journal.ppat.1010777 (PMC9980815; doi:10.1371/journal.ppat.1010777)
Supplement: S1 Methods — (DOCX) [file ppat.1010777.s006.docx]

**Supporting Information for**

Wbm0076, a candidate effector protein of the *Wolbachia* endosymbiont of *Brugia malayi*, disrupts eukaryotic actin dynamics

Michael K. Mills^1^, Lindsey G. McCabe^1^, Eugenie M. Rodrigue^1^, Karl F. Lechtreck^2^, and Vincent J. Starai^1,3^

Departments of ^1^Microbiology, ^2^Cellular Biology, and ^3^Infectious Diseases

University of Georgia, Athens, GA

To whom correspondence should be addressed:

Vincent J. Starai, (706) 542-5755, [vjstarai@uga.edu](mailto:vjstarai@uga.edu)

**Supporting Materials and Methods**

**Phalloidin staining of the yeast actin network.** Yeast strains harboring a pYES2/NT A control plasmid, or a pYES2/NT A plasmid cloned with one of the following: *w*Bm0076, *w*Bm0076 (W280A), *w*Bm0076 (R258A), were grown overnight in CSM medium lacking uracil. Cells were subcultured to fresh CSM-Ura and outgrown for 2h at 30°C with shaking. To induce *w*Bm0076 expression, 1 μM β-estradiol was added and continued shaking for 5h at 30 °C. Formaldehyde was added to a final concentration of 4% (v/v) and incubated for 10 min at room temperature. Cells were harvested by centrifugation (2000 x *g*, 5 min), suspended in 1 volume 0.1M KP_i_ buffer containing 4% formaldehyde, and incubated at room temperature with agitation. Cells were harvested by centrifugation and suspended in 1 volume 0.1M KP_i_ containing 10 mM ethanolamine. After 10 min incubation at room temperature, cells were suspended in 1/30 volume 0.1 M KP_i_ and stored at 4°C.

To stain the actin cytoskeleton, approximately 10^6^ cells for each condition were harvested via centrifugation and suspended in 25 µl 3.3 µM rhodamine phalloidin (Molecular Probes), which was previously suspended in PBS containing 0.1% Triton X-100 (v/v). Cells were incubated at room temperature for 30 min in the dark with agitation, harvested, washed 3 times with 500 µl PBS, then imaged.
