# Supplementary material for: Wbm0076, a candidate effector protein of the Wolbachia endosymbiont of Brugia malayi, disrupts eukaryotic actin dynamics
Source: PLoS Pathog. 2023 Feb 17;19(2):e1010777. doi: 10.1371/journal.ppat.1010777 (PMC9980815; doi:10.1371/journal.ppat.1010777)
Supplement: Attachment — Submitted filename: PLOS Pathogen rebuttal.pdf [file ppat.1010777.s011.pdf]

In this supplemental file, we provide a point-by-point response to each of the previous reviewers' comments regarding our original manuscript previously entitled, "Wbm0076, a candidate effector protein of the *Wolbachia* endosymbiont of *Brugia malayi*, disrupts eukaryotic actin dynamics" We thank the reviewers for their insightful comments, and we appreciate the opportunity to respond to these comments. Below, please find responses to each reviewer's comments (starting with >); our responses are prefaced with "**Authors' response:**":

Reviewer 1:

Wolbachia pipientis is an important and ubiquitous symbiont in insects, nematodes, and arthropods more broadly. We know from work in the field that Wolbachia expresses a type IV secretion system and that it likely uses its secreted effectors to modify host biology. Understanding the evolution and function of these effectors is important and here, the authors follow up on their large-scale yeast screen identifying candidate effectors in the Wolbachia that infect the nematode Brugia malayi. They show that one protein from the Wolbachia from Brugia (wBm0076) co-localizes with a couple of actin binding proteins (Abp1p and Abp140) and alters vesicle trafficking dynamics in the cell. I was a bit confused by the title as it downplays the vesicle work, and the authors don't ever actually show the actin cytoskeleton of the yeast are altered by the presence of wBm0076 but instead focus on Abp1p as a proxy for actin dynamics. It would've been nice to see some basic phalloidin staining. Overall, I think this is a nice study, but I have a few suggestions for the authors below. Additionally, there were many typos throughout with regards to figure citations and the use of correct nomenclature for Wolbachia proteins. I suggest they carefully read through the manuscript before resubmission.

Major revisions

>Figure 2: As the authors note in the text (lines 198-200), it seems like the biggest phenotype is the large number of Abp1-mCherry foci per cell - this should be quantified, and statistical differences noted.

Author's response: We agree that one of the most striking phenotypes induced by *w*Bm0076 expression in yeast is the increase in Abp1-positive punctae. In our previous work (Carpinone *et al*., 2018, *PLoS One*), we have already quantified this observed increase in Abp1p foci during the expression of *w*Bm0076 in yeast cells. Importantly, the primary focus of Figure 2 is determining the colocalization of known endocytic markers (Ede1p, Sla1p, Sac6p) with the well-known actin patch marker, Abp1p, in the presence or absence of *w*Bm0076 expression. Therefore, we have chosen to not re-analyze the statistics of Abp1p punctae formation under these conditions.

>Line 295 "its ability to produce aberrant branched actin structures is dependent on its conserved VCA subdomains (Fig 2)" - do you mean Figure 3? Quite frankly, I do not think that Figure 3 shows this - there is no actin staining and Abp140-GFP puncta are not quantified. Why use Abp140GFP as a proxy for actin dynamics? Why switch from

Abp1p? Why not stain actin?

Author's response:

We apologize for the mistaken switching of Figs. 2 and 3 in our original submission. We have now placed the correct figure (Fig. 3) in this results section. Abp140p-GFP has routinely been used as a marker for both actin patches and cables (Doyle and Botstein, 1996, *PNAS*). For the experiment performed in Fig. 3, we utilized a mRuby2-derivative of Wbm0076, which prevented us from using an additional red-fluorescent protein (Abp1-RFP). As Abp140-GFP and Abp1-RFP both stain branched actin structures in the cell, we found it acceptable to utilize Abp140-GFP for this experiment. Nevertheless, we now complement these data with phalloidin staining of the entire yeast actin network in Supplemental Figure S2, which shows the loss of actin cables in response to the presence of Wbm0076, and the increase of phalloidin-positive punctae. While we were not able to observe the presence of remaining actin cables in the VCA mutant constructs of *w*Bm0076-expressing strains, it is well-known that observing cables in yeast via phalloidin staining is rather difficult due to the overall weak staining of yeast actin cables.

>I love Figure 4 - the complementation of toxicity with LactC2 is striking! I wonder, do the WH2, central, and acidic mutants show different localization than WT with regards to co-localization of Abp1p? Similarly, I wonder if you could quantify Abp1pRFP puncta in this background compared to vector alone (as requested above).

Author's response:

We appreciate the reviewer's kind words regarding this experiment. The primary goal of this experiment is to show that the membrane localization of Wbm0076 is required for its ability to induce the formation of the Abp1-positive branched actin structures. While it would be interesting to measure the number of Abp1-positive punctae in the domain-mutated *w*Bm0076-expressing strains, the results of this particular experiment would not lend additional information towards the discovery that the LactC2 phosphatidylserine-binding domain restores both the colocalization of Wbm0076 with Abp1p-positive actin patches, and the toxicity of *w*Bm0076 expression. Furthermore, repeating these particular experiments with a full panel of mutant *w*Bm0076 strains is exceedingly difficult due to the lack of financial resources and dedicated personnel.

>With regards to Figure S3 - the western has two band sizes here - what is that lower band? The TMWbm0076 does indeed look to have less expression when compared to the other columns - where is your loading control? Would be good to add because as it is, it seems like your reduced toxicity could be linked to reduced.

<u>Author's response:</u>

We have repeated this western blot to include the visualization of a yeast constitutively-expressed loading control, Sec17p. In this new figure, the presence of alternatively-sized bands are rather limited, suggesting the initial figure submitted contained significant degradation products of our detected proteins. We now see that most *w*Bm0076-containing protein products are expressed at near-equivalent levels.

<u>Minor revisions</u>

>: Line 83-85: is it Wolbachia secreted proteins (wSP's) or Wolbachia surface proteins? Pick one please.

<u>Author's response:</u>

We apologize for the confusion. The first use of *w*SP in lines 86-87 was meant to describe *Wolbachia* surface proteins and we have now kept it as such (line 84). The further use of "*w*SP" in our manuscript is considered incorrect and should refer to the Type IV-secreted effector proteins of *Wolbachia*. We have changed each incorrect use of "*w*SP" to (candidate) Type IV-secreted effectors, or some derivative of that phrase, throughout the document.

>Line 110-112 - I would say this work is excellent genetic evidence that Wolbachia encodes a protein that modulates the actin cytoskeleton. The production and secretion and the "critical" - not so much supported by these data. You can speculate only.

<u>Author's response:</u>

We agree with the reviewer's comments here that the data only provide additional evidence to previous work that speculate that *Wolbachia* produces and secretes proteins that modulate the actin cytoskeleton. We do think the protein is <u>likely</u> critical for *Wolbachia* mobilization and we intended the language to be speculative. We now state that "…which may be important for the previously-proposed ability of *Wolbachia* to mobilize through host cells via cell-to-cell transmission pathways" (lines 112-113).

>Line 221 - you mean Fig S3 here - please check throughout the manuscript to ensure you refer to the correct figures and data files.

<u>Author's response:</u>

We have now made sure to correctly refer to the proper figures throughout the document.

>I suggest adding a citation to Figure 6 to line 319 as well.

Author's response:

We have added this citation.

>Line 362 - should be wBm0076 (not Wbm0076) - check throughout - saw this also on line 293.

Author's response:

When speaking about the gene throughout the document we utilize the term "*w*Bm0076" and when speaking about the expressed protein, we use the term "Wbm0076". We have gone through the document to be sure that the use of *w*Bm0076 or Wbm0076 is appropriate.

>Line 335 - actin has also been shown to be important for Wolbachia's maternal transmission in fruit flies (see Sheehan et al., 2016 and Newton et al., 2015).

Author's response:

We have made sure to highlight the importance of host actin dynamics for the proper localization and transmission of *Wolbachia* in *Drosophila* (lines 114-115).

Reviewer 2:

This study is a continuation of the author's previous work on the interaction of Wolbachia endosymbiont of Brugia malayi proteins with actin and membrane-interacting proteins of yeast, Saccharomyces cerevisiae. The said proteins cannot be easily studied in the native Wolbachia-worm symbiosis, as both organisms are genetically intractable. Previous work has already established the toxicity of the putative Wolbachia effector, Wbm0076, in yeast cells and suggested that the interaction of this heterologously expressed protein with the membrane might lead to cell lysis. This manuscript provides a genetic dissection of the mechanism of toxicity of Wbm0076, and postulates that the phenotype observed in yeast (cell killing) can have a different manifestation in the native host (promoting of Wolbachia endocytic uptake by host cells). Yest genetics experiments are rigorous, they include all required controls.

Major issues

The conclusions about Wbm0076 toxicity, its interactions with actin and endocytosis machinery are well validated, both here and in Carpinone et al. 2018.

I have three clarification requests, mostly considering data interpretation and importance of Wbm0076 in the native Brugia-Wolbachia symbiosis:

>1. What is the timeline of events here? How long after Wbm0076 interaction with endocytic machinery do yeast cells die?

Author's response:

While we have not precisely measured the timeline of Wbm0076 interactions with endocytic machinery and the exact time of yeast death, we feel that defining this timeline is beyond the scope of this work. As we are overproducing Wbm0076 at levels far beyond what would normally be experienced by the physiologically-relevant host cell during the *Wolbachia*:*Brugia* endosymbiosis, we feel that understanding the kinetics of these interactions and cell death in yeast is somewhat irrelevant. What we have observed (not quantified) from our previous work, however, is that yeast cells expressing *w*Bm0076 are usually lysed at timepoints later than 6 hours post-induction (between 6 and 16 h) (Carpinone *et al*, 2018).

>2. Does interaction of Wbm0076 with membrane causes cell puncture at the site of interaction?

Author's response:

We have not yet explored the cell lysis phenotype induced by *w*Bm0076 expression in yeast. Although this is an intriguing question, we did not pursue this study as we hypothesize that the cell puncture/lysis phenotype is a result of the loss of cellular monomeric actin induced by the hyperactivation of Arp2/3 complex via Wbm0076 activity. As these strains lose observable actin cables/F-actin structures due to the altered polymerization event (Fig. S2, Carpinone *et al.*, 2018, *PLoS One*), we anticipate that this lysis phenotype mirrors the lysis phenotype observed in growing yeast strains that have been depleted for F-actin (Sahin, *et al.,* 2008, *PLoS One*). Furthermore, Wbm0076 accumulates at several sites around the plasma membrane (this work and our previous work), yet observation of yeast cells lysing under these conditions appear to show a break at a single point in the cell wall in what appears to be only budding cells (Carpinone, *et al.*, 2018, *PLoS One*, Figure 4). Despite these anecdotal statements, it remains possible that association of Wbm0076 with the plasma membrane induces cell lysis at that distinct point. The foundation of this work is to show that Wbm0076 disrupts actin dynamics via a WAS(p)-like activity, however, and not the ability of Wbm0076 to lyse cells.

>3. The reasoning behind toxicity in yeast turning into endocytosis promoting-phenotype in native Wolbachia hosts is unclear to me. What is the "physiological" reason for yeast cell death here? Do they starve as a result of endocytic pathway disruption? And how do they die, is it cell lysis or, eg. apoptosis?
Also, in the endocytosis-promoting function, Wolbachia would be outside of the host cell, where would the Wbm0076 be? On its surface? Or in the host cell already?

I am asking as the cell killing caused by Wbm0076 can be interpreted differently. As Brugia malayi cannot survive without Wolbachia, the effector studied here could be a Wolbachia life-insurance: killing of Wolbachia cells could release this effector and kill host cells. Thus, under normal conditions Wbm0076 could be strictly intracellular. As this is a much simpler conclusion, and it seems to fit with the data presented here better, I wonder whether it is a possibility in this system.

Another option is consistent with the interpretation provided here (the importance of Wbm0076 in cell-to-cell movement of Wolbachia) but suggests that the Wbm0076 would enable Wolbachia exit from already infected cells rather that its subsequent uptake. Studying the native distribution of Wolbachia in Brugia seems important to discern between these two options. This seems especially important as authors postulate conservation of endocytic machinery between all eukaryotes (hence the heterologous system usage) yet predict a completely different interaction outcomes between the model used here and the native Wolbachia hosts.

Author's response:

We appreciate the reviewer's interest in this question, as it remains a completely unstudied topic due to the difficulty of working in the *B. malayi:Wolbachia* system at the molecular level with current technologies.

In our response above and in our manuscript, we state our hypothesis that yeast lysis and death is due to the ability of Wbm0076 to disrupt actin polymerization dynamics. As actin is required for endocytosis, the loss of normal actin dynamics results in the concomitant loss of endocytosis. To the best of our knowledge, yeast strains which are defective for endocytosis (an *end4^{ts}* strain, for example) do not grow at a non-permissive temperature. However, these strains have not been noted to extensively lyse under these conditions (A. Wesp, *et al.,* 2017, *Mol Biol Cell*). Therefore, we do not anticipate that the lysis phenotype in wBm0076-expressing strains is due to the loss of endocytosis, but rather due to the loss of F-actin. We have used the measure of yeast endocytosis as a tool to observe the effects of Wbm0076 on cellular actin dynamics.

It is true that *B. malayi* undergoes extensive apoptosis during development when *Wolbachia* is cleared from the nematode via antibiotic treatment (Landmann, *et al.,* 2017, *PLoS Path*). In these experiments, the authors treated infected jirds (the laboratory host for *B. malayi*) with antibiotics for 6 weeks. *B. malayi* was harvested from

these animals 8 weeks later, and assayed for apoptotic nuclei. While the researchers observed a 99% elimination of *Wolbachia* from both adult worms and L4-stage worms, only about 22% of the measured female worms showed extensive apoptosis in germline cells. In addition, 83% of intrauterine microfilariae isolated from treated females were undergoing apoptosis, and apoptotic cells appeared to accumulate over time during microfilariae development. Therefore, the apoptosis observed in these nematodes is not an acute event induced by an immediate bacterial response to antibiotics. Strikingly, despite being localized in the lateral chord of male nematodes - rather distant from the gonads - male nematodes isolated from antibiotic-treated jirds were found to have apoptotic cells in their germline cells, suggesting that *Wolbachia* can prevent apoptosis in male worms, despite being localized at sites distant from the cells undergoing apoptosis (Landmann, *et al.,* 2017, *PLoS Path*). Finally, it is known that *w*Bm0076 transcription levels are highest in L4 and adult worms, which does not coincide with the apoptosis observed in juvenile microfilariae which lack *Wolbachia* (lines 372-374). Therefore, it is <u>extremely</u> unlikely that *Wolbachia* induces cell lysis in the host through a 'suicide' mechanism involving Wbm0076 translocation into host cells. In light of all of these facts, we do not feel comfortable making this possibility a statement in our discussion section.

It is well known that many bacteria modulate actin dynamics of host cells to promote its uptake through the use of secreted effectors (for example, *Shigella*; Kühn, *et al*., 2020, *Cell Rep*). With this in mind, *Wolbachia* has been observed in the pseudocoelomic space (extracellular), where it interacts with the distal tip of nematode ovaries (lines 373-374, Landmann *et al*., 2012, *Biol Open*). In this case, *Wolbachia* must find its way into cells, which could be due to an actin-driven endocytic-like uptake process. As we now show that Wbm0076 appears to behave like a WAS(p)-family protein capable of modulating actin polymerization via potential Abp1p interactions (Fig. 5), it is certainly within the realm of possibility that the physiologically-relevant concentration of Wbm0076 provided by *Wolbachia* (and along with other Type IV-secreted effectors) properly regulates host actin dynamics for endocytic uptake of *Wolbachia*.

We completely agree with the reviewer's statement that *Wolbachia* may use Wbm0076 activity to exit host cells and to promote its cell-to-cell movement in the nematode, which we state in lines 364-385. We also agree with the reviewer that in order to fully understand the role that Wbm0076 plays in the endosymbiosis, localization of Wbm0076 in the natural host (bacterial surface? Host cell membranes?) will absolutely be an important experiment to carry out in the future (lines 401-404).

<u>Minor issues</u>

No minor issues, the data are beautifully presented, and the text is well written.
